# Probiotic *Lactobacillus fermentum* strain JDFM216 stimulates the longevity and immune response of *Caenorhabditis elegans* through a nuclear hormone receptor

**DOI:** 10.1038/s41598-018-25333-8

**Published:** 2018-05-10

**Authors:** Mi Ri Park, Sangdon Ryu, Brighton E. Maburutse, Nam Su Oh, Sae Hun Kim, Sejong Oh, Seong-Yeop Jeong, Do-Youn Jeong, Sangnam Oh, Younghoon Kim

**Affiliations:** 1 Department of Animal Science and Institute of Milk Genomics, Chonbuk National University, Jeonju, 54896 Korea; 2 R&D Center, Seoul Dairy Cooperative, Ansan, Gyeonggi 15407 South Korea; 3 Department of Biotechnology, College of Life Sciences and Biotechnology, Korea University, Seoul, 02841 South Korea; 4 Department of Animal Science, Chonnam National University, Gwangju, 61186 Korea; 5 Microbial Institute for Fermentation Industry, Sunchang, Jeonbuk 56048 Republic of Korea; 6 Department of Functional Food and Biotechnology, Jeonju University, Jeonju, 55069 Republic of Korea

## Abstract

Here, we examined the functionality of *Lactobacillus fermentum* strain JDFM216, a newly isolated probiotic bacterium, using a *Caenorhabditis elegans* model. We determined bacterial colonization in the intestinal tract of *C. elegans* by plate counting and transmission electron microscopy and examined the survival of *C. elegans* using a solid killing assay. In addition, we employed DNA microarray analysis, quantitative real time-polymerase chain reaction, and immunoblotting assays to explore health-promoting pathways induced by probiotic bacteria in *C. elegans*. Initially, we found that the probiotic bacterium *L. fermentum* strain JDFM216 was not harmful to the *C. elegans* host. Conditioning with JDFM216 led to its colonization in the nematode intestine and enhanced resistance in nematodes exposed to food-borne pathogens, including *Staphylococcus aureus* and *Escherichia coli* O157:H7. Interestingly, this probiotic strain significantly prolonged the life span of *C. elegans*. Whole-transcriptome analysis and transgenic worm assays revealed that the health-promoting effects of JDFM216 were mediated by a nuclear hormone receptor (NHR) family and PMK-1 signaling. Taken together, we described a new *C. elegans*-based system to screen novel probiotic activity and demonstrated that preconditioning with the probiotic *L. fermentum* strain JDFM216 may positively stimulate the longevity of the *C. elegans* host via specific pathway.

## Introduction

Probiotic bacteria are defined as living microorganisms that exert beneficial effects on human health when ingested in sufficient quantity^1^; these effects include improved intestinal microbial balance, immunomodulation, and extended life span^2,3^. The anti-aging effects of probiotics were first proposed by Metchnikoff who reported that Bulgarian farmers consuming large quantities of fermented milk containing lactobacilli experienced enhanced health and longevity^4^. To date, many research groups have actively investigated novel probiotics and the functional characteristics of potential probiotic candidates. Unfortunately, studies on the anti-aging influence of probiotics are limited because of a lack of suitable experimental models to verify host longevity. Mechanisms underlying the probiotic effects of bacteria are also unclear.

In this respect, studies on *Caenorhabditis elegans* have provided valuable insights into the functional aspects of anti-aging and innate immunity and have led to the exploration of the multiple genetic pathways that determine longevity^2,3,5^. In particular, the nematode *C. elegans* is a suitable experimental model because its life span is influenced by host defense mechanisms that are well characterized and its metabolism has been highly conserved throughout evolution. Moreover, facile genetic manipulation can be performed in *C. elegans* and it can be grown in large quantities, providing the opportunity to investigate structures, biosynthesis, and functions of microbe-associated molecular patterns (MAMPs) in a relatively simple model system^6^. Importantly, innate immunity is an evolutionarily conserved response to pathogens and is the first line of defense in most organisms. Several conserved signaling pathways that function in the perception of and defense against bacterial pathogens have been identified in *C. elegans*. These pathways include the NSY-1/PMK-1 MAP kinase signaling pathway, the DAF-2/DAF-16 insulin/insulin-like growth factor-1-like signaling pathway, and the BAR-1 β-catenin signaling pathway^7–9^. Although many conserved innate immune components have been identified in *C. elegans* using genetic and biochemical approaches, extensive characterization of the probiotic-regulated signaling networks in host responses and outcomes of infections during aging in *C. elegans* are required.

The nuclear hormone receptor (NHR) superfamily is a class of ligand-activated transcription factors regulated by small lipophilic hormones. When activated, they stimulate or repress target gene transcription. Even small differences in ligand structures may result in dramatic changes in transcriptional activity and specificity, such as changes in the control of diverse aspects of metabolism, development, and aging^10^. However, the endogenous ligands of many NHRs remain poorly characterized, partly because ligands constitute very minor components of highly complex animal metabolomes^11^. Although 284 genes in the *C. elegans* NHR superfamily have been identified, approximately 20 of them have been genetically analyzed^12^.

In this study, we focused on the probiotic *Lactobacillus fermentum* strain JDFM216 (referred to as JDFM216) and screened its beneficial effects, including its resistance to acid and bile, mucin attachment, and lowering of cholesterol levels, using *in vitro* assays. We investigated on the anti-aging and immune-modulating effects of JDFM216 in *C. elegans* as potential probiotic bacterium and identified on underlying mechanism involves NHRs using whole transcriptome analysis.

## Results

### JDFM216 extends *C. elegans* life span

**Figure 1:**
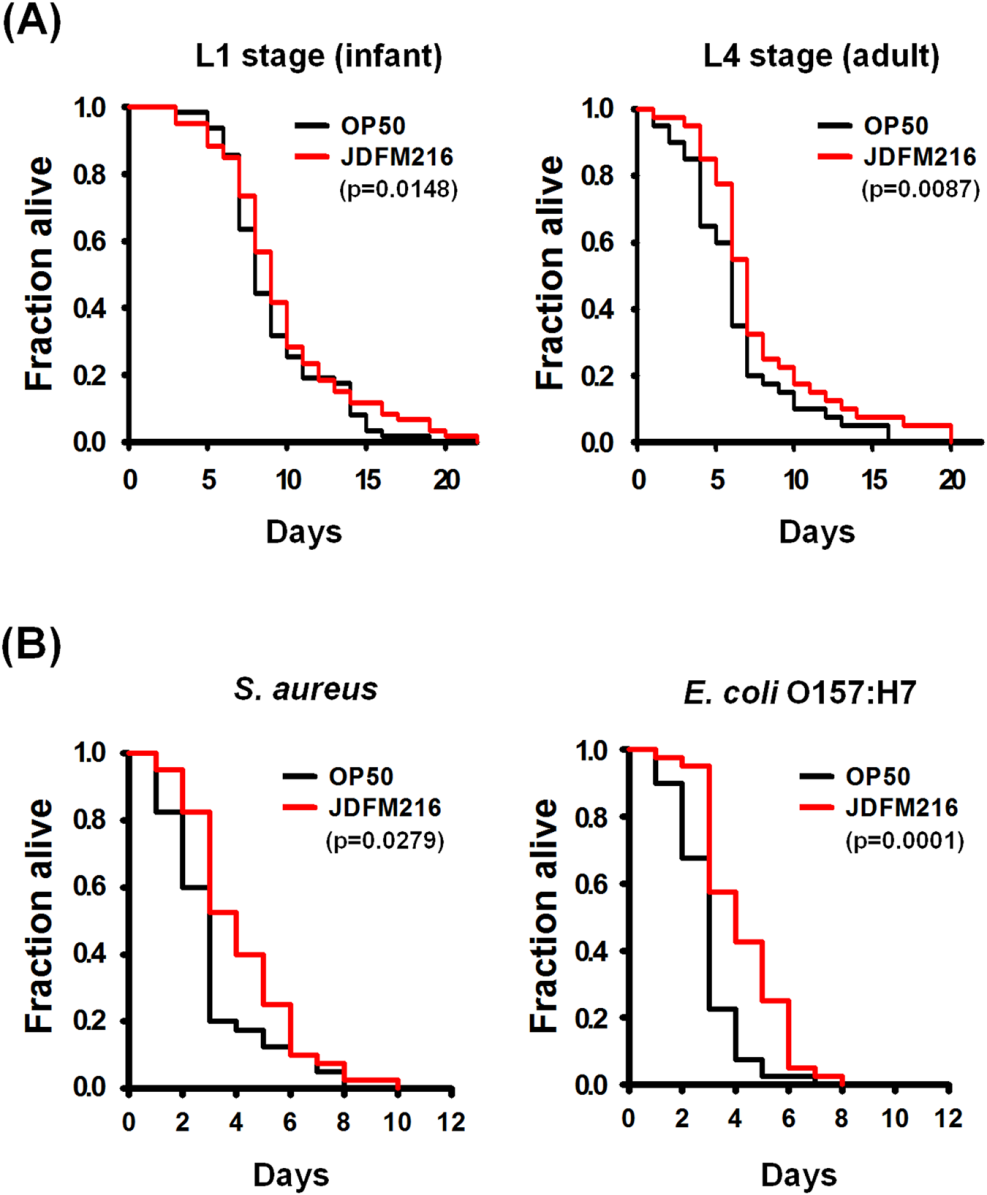
(**A**) Enhanced lifespan of *C. elegans fer-15;fem-1* via conditioning with *Lactobacillus fermentum* strain JDFM216 for 24 h prolonged the lifespan of *C. elegans* nematodes. Survival statistics: *p* = 0.0148 for L1 stage (left), and *p* = 0.0087 for L4/young adult stage (right) of nematodes compared with worms feeding on the *E. coli* OP50 control strain (n = 30 per plate). (**B**) Immune stimulation of *C. elegans* via pre-conditioning with JDFM216 for 24 h prolonged the lifespan of *C. elegans* strains, N2 wild-type or pmk-1 mutant infected with *S. aureus* strain RN6390. Survival statistics: *p* = 0.0026 for 1 day, *p* = 0.0492 for 3 days, *p* = 0.0000 for 6 days, and *p* = 0.0103 for 9 days conditioned nematodes compared with worms feeding on the *E. coli* OP50 control strain (n = 30 per plate).

In a preliminary study, we selected JDFM216 based on of their human origin, nonpathogenic status, confirmation of their ability to survive during gastrointestinal transit (acid tolerance/bile resistance) using *in vitro* tests, intestinal mucin adhesion properties, and antimicrobial activities (Fig. S1 and Table S1) and analyzed its genetic features^13^. These studies demonstrated that JDFM216 exhibits probiotic characteristics, and it hypothesized that JDFM216 would confer beneficial health effects to the host. Even though aging is much more than a lifespan measurement, lifespan has been extensively employed for measuring aging development. Thus, *C. elegans* is one of the suitable model systems for aging research since they provide simple and convenient methodology to determine lifespan extension at the molecular levels^14^. Thus, we first examined whether age affects the life span-prolonging effect of JDFM216 in *C. elegans*. L1/larval-stage and L4/young adult-stage worms grown on NGM plates seeded with OP50, individually transferred to plates containing OP50 or JDFM216, and then maintained at 25 °C. In the present study, we confirmed that the probiotic worms fed with JDFM216 displayed significantly prolonged life span at both L1 and L4 stages compared with worms fed with OP50 (normal feeding bacteria) that were used as a control (p = 0.0148 for L1 stage and p = 0.0087 for L4/young adult stage) (Fig. 1A). Enhanced life spans were more evident in L4/young adult-stage worms. Importantly, similar results have been obtained by Ikeda *et al*.^2^ who have demonstrated that *C. elegans* fed with selected lactobacilli have an increased average life span ranging from 17% to 33%. Therefore, this result indicated that conditioning with JDFM216 significantly influenced the longevity of *C. elegans in vivo*.

### JDFM216 enhances resistance in *C. elegans* against pathogen infection

Next, to estimate whether the probiotics influenced the host resistance to infection by food-borne pathogens, agar-based solid killing assays were performed according to previously described methods with slight modifications^15,16^. Nematodes were conditioned by transferring *fer-15;fem-1* L4/young adult worms to a JDFM216 lawn for 1 day and then exposing them to *S. aureus* RN6390 or *E. coli* O157:H7 EDL933, food-borne pathogens that also kill *C. elegans* via an infection-like process in the intestine. As expected, worms were killed within 10 days after transferring to a lawn containing either pathogen. The viability of *C. elegans* conditioned with JDFM216 for 24 h was significantly enhanced compared with that of control worms pre-exposed to the nonpathogenic *E. coli* strain OP50 (p = 0.0279 for *S. aureus* and p = 0.0000 for EDL933) (Fig. 1B). These results are in agreement with the pathogen-protective effect of *L. acidophilus* demonstrated by our group^17^ and reports indicating that specific lactic acid bacteria can enhance defense and stress resistance in *C. elegans*^5^. It has recently been reported that *L. fermentum* produces inhibitory compounds, including H_2_O_2_, bacteriocin, and biosurfactants, to inhibit the growth of intestinal and food-borne pathogens. Moreover, an emerging clinical study has shown that *L. fermentum* is effective in decreasing intestinal pathogens and increasing the ratio of probiotic bacteria in healthy humans^18^. Taken together, the results indicate that conditioning with JDFM216 makes *C. elegans* resistant to infection by *S. aureus* and *E. coli* O157:H7.

### JDFM216 attach to the intestinal tract of *C. elegans*

**Figure 2:**
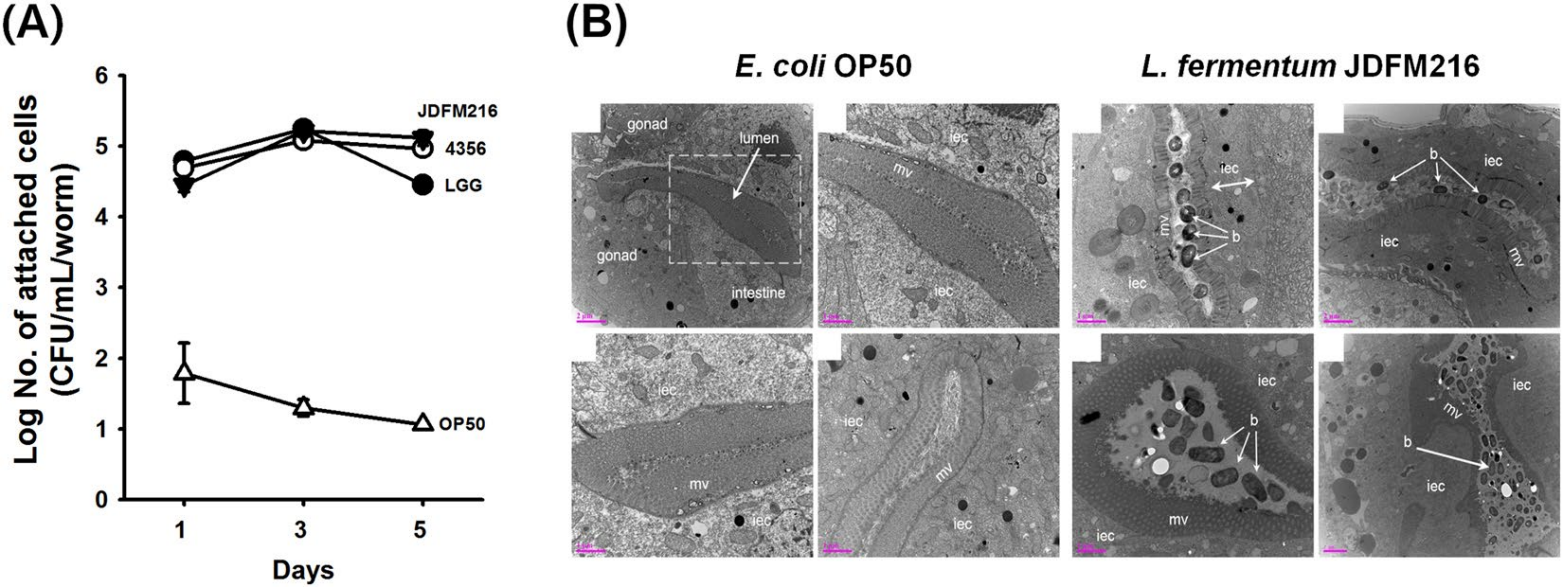
(**A**) Plate counting and (**B**) transmission electron microscopy (TEM) images indicated that the *Lactobacillus fermentum* strain JDFM216 was highly attached on the *C. elegans* intestine compared with *E. coli* OP50. Scale bar is 2 μm.

As noted above, many different criteria have been used to select potential probiotic strains. Of these, probiotic attachment to intestinal epithelial cells and colonization of the intestinal tract are the most important factors because they affect the ability of the bacterial species to function as a probiotic and also maintain the normal gut microbiota balance in the host^19^. Importantly, *C. elegans* intestinal cells are similar in structure to those of humans and are the principal cells involved in the host’s defense mechanisms by functioning as immune cells and a mucosal defense barrier^3^. Thus, we postulated that bacteria present in the *C. elegans* intestinal lumen accelerate health-promoting effects during the attachment process by activating defense mechanisms in the host. Therefore, we evaluated the ability of JDFM216 to attach to the *C. elegans* intestinal tract on days 1, 3, and 5. After exposure to JDFM216, *C. elegans* were scored according to the degree of bacterial accumulation within the intestinal lumen. As shown in Fig. 2A, JDFM216 exhibited significantly high colonization ability. We found that in the *C. elegans* intestine, JDFM216 maintained a high persistence of >4 CFU/mL per worm for 5 days, similar to that of other probiotic strains, including *L. rhamnosus* strain GG and *L. acidophilus* ATCC 4356, as positive controls (Fig. 2A). It also supports the finding that JDFM216 remained in the intestine of *C. elegans* after transferred to OP50 (Fig. S2). Next, we used TEM to visualize and verify our findings. TEM of worms allowed a high resolution of the *C. elegans* intestinal lumen (Fig. 2B). Consistent with plate counting, TEM images demonstrated that numerous large pockets of undigested JDFM216 cells accumulated in the intestinal lumen as clumps of cells surrounded by extracellular matrix and that the worm’s intestine was completely filled with bacteria (Fig. 2B). In contrast, negative control OP50-fed worms exhibited no clump of bacterial cells and no distended intestinal lumen or intestinal epithelial cell filled with lipid droplets. These results suggested that compared with normal feeding bacterium OP50, JDFM216 can attach to the *C. elegans* intestine, as determined by plate counting results and TEM images. Taken together, the results indicated that JDFM216 had host health-promoting effects, including longevity and resistance to pathogenic infection, as well as demonstrated excellent colonization of the worm intestinal tract.

### Enhanced longevity and immunity of *C. elegans* fed with JDFM216 are mediated by PMK-1 signaling

**Figure 3:**
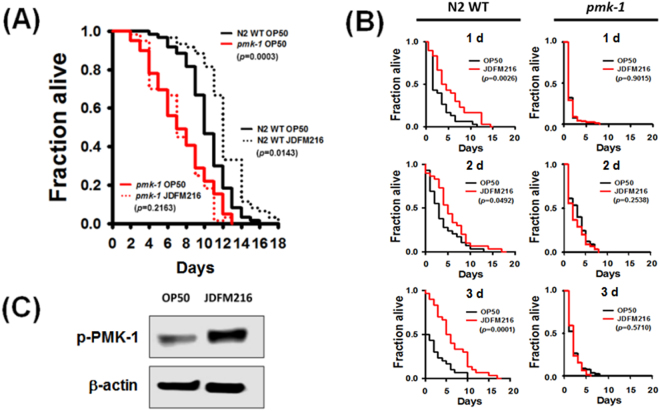
(**A**) PMK-1 signaling regulate the lifespan of *C. elegans* (Survival statistics: *p* = 0.0003 for *pmk-1* mutant nematodes compared with worms feeding on the *E. coli* OP50 control strain (n = 30 per plate). (**B**) Survival rate of wild-type N2 (left column) or *pmk-1* mutant (right column) via pre-conditioning with JDFM216 for 1, 2, and 3 days prolonged the lifespan of *C. elegans* nematodes against *S. aureus* RN6390. Survival statistics: *p* = 0.0026 for 1 day, *p* = 0.0492 for 2 days, or *p* = 0.0001 for 3 days in N2 worms and *p* = 0.9015 for 1 day, *p* = 0.2398 for 2 days, or *p* = 0.5710 for 3 days in *pmk-1* worms compared with worms feeding on the *E. coli* OP50 control strain (n = 30 per plate). (**C**) Immunoblot analysis of PMK-1 activation in whole worm lysates of worms by JDFM216 using the phosphorylated PMK-1 (p-PMK-1). β-actin was used as a loading control. The blots were cropped and full length blots are presented in Supplementary Figure S3.

In *C. elegans*, the conserved PMK-1 p38 mitogen-activated protein kinase pathway plays a role in regulating longevity and innate immunity genes. In particular, PMK-1 mediates host defense in *C. elegans* by regulating the expression of genes encoding proteins important for the response to infection by a diverse range of pathogenic bacteria^7,16,20,21^. Therefore, we hypothesized that JDFM216 enhances host response associated with aging by stimulating the immune system, including PMK-1 signaling. Initially, we performed solid killing assays using *pmk-1* mutant or N2 wild-type worms to confirm whether the PMK-1 signaling pathways were directly involved in the JDFM216-induced longevity of *C. elegans*. Consistent with previous results^22^, we confirmed a significant decline in the life span of *pmk-1* mutants compared with that of N2 wild-type nematodes fed with OP50 (p = 0.0003 for *pmk-1* mutants fed with OP50 versus N2 wild-type nematodes fed with OP50) and observed an enhanced life span of *C. elegans* N2 wild-type exposed to JDFM216 (p = 0.0143 for N2 wild-type nematodes fed with OP50 versus N2 wild-type nematodes fed with JDFM216; Fig. 3A). Surprisingly, *pmk-1* deletion resulted in even lower survival despite being exposed to JDFM216 that enhanced the life span of *C. elegans* N2 wild-type (p = 0.2163 for *pmk-1* mutants fed with OP50 versus *pmk-1* mutants fed with JDFM216). Importantly, these results were consistent with previous data that the lifespan of *pmk-1* mutant was significantly shorter than N2 feeding on OP50^23^. Taken together, our results indicated that *pmk-1* signaling contributed to longevity and immunity and that JDFM216 may stimulate the *pmk-1* pathway to enhance the immune response and longevity of the host.

Next, we explored the change in innate immunity during aging by investigating the susceptibility of *C. elegans* laboratory wild-type strain N2 or *pmk-1* mutant to *S. aureus* RN6390 after exposure to JDFM216 or OP50. We exposed *C. elegans* to JDFM216 at the L4/young adult stage for 1, 2, and 3 days and then transferred them to plates seeded with *S. aureus* RN6390. We observed that *C. elegans* preconditioned with JDFM216 demonstrated improved survival rates after transferring to *S. aureus* regardless of age (Fig. 3B). As expected, JDFM216 in the N2 wild-type nematode intestine strongly enhanced resistance in nematodes exposed to *S. aureus* (p = 0.0026 for day 1, p = 0.0492 for day 2, and p = 0.0001 for day 3) compared with each corresponding OP50 control. Compared with OP50, JDFM216 did not increase the life span of *pmk-1* mutants of different age groups (p > 0.05). To ascertain whether the activity of PMK-1 is directly stimulated by JDFM216, we determined the level of phosphorylated PMK-1 by using a specific antibody that recognizes the phosphorylated form of PMK-1. Our results showed that the phosphorylation levels of PMK-1 were critically upregulated in *C. elegans* with JDFM216, as compared with OP50 control (Fig. 3C). These results indicated that JDFM216 effectively stimulated PMK-1 signaling pathways involved in the longevity and immunity of *C. elegans*.

### NHR family is involved in the longevity and immune response of *C. elegans* with PMK-1 signaling

In addition to PMK-1 signaling, we sought to identify the genetic determinants involved in the mechanism underlying improvement in longevity and host response with age. Previously, microarray-based gene expression studies on aging *C. elegans* revealed numerous changes in gene expression during the aging process^24,25^. Thus, we explored newly identified genes specific to longevity and host defense mechanisms of *C. elegans* via JDFM216 using whole-transcriptome analysis. Preconditioning with JDFM216 for 24 h stimulated specific gene regulation of multiple receptors. Compared with OP50, the transcription of many differentially expressed genes was found to be regulated by JDFM216. In total, 163 genes were upregulated and 29 were downregulated >5-fold when *C. elegans* were preconditioned with JDFM216. These genes may be involved in several functions, including biological processes (cell communication, neuron migration, defense response, and development) and molecular function (DNA binding) based on the known function of the corresponding gene product or by homology with previously described protein functions as determined from the KEGG gene database. Among these, a large portion of differentially expressed genes are those associated with C-type lectin family members, including *clec-60* (375.88-fold) and *clec-78* (58.48-fold), and F-box protein-related genes (5.22- to 6.45-fold). Our results suggested that several C-type lectin and F-box proteins are involved in the life span of *C. elegans* under stress conditions by influencing the longevity of nematodes^22,26^. Expression profiling studies have revealed that NHR family genes are among the genes most highly induced by JDFM216 (Table 1). Indeed, there is evidence of a role of NHR in *C. elegans* longevity^27^. Previously, microarray expression analysis examining the response of *C. elegans* to nutrition has demonstrated that *nhr-49* plays a key role in nutritional signaling, enabling the induction of β-oxidation genes^28^. Taken together, this activation of NHR family genes that are inducible by JDFM216 suggested that they may play a role in longevity mechanisms.

**Table 1:** List of differentially expressed genes (>5.0 folds) in *Caenorhabditis elegans fer-15;fem-1* worms fed with *Lactobacillus fermentum* strain JDFM216 for 24 h compared to *Escherichia coli* OP50 fed control.

Group and gene	Gene number	Fold change	Description
**C-type lectin-related**			
*clec-60*	ZK666.6	513.04	C-type LECtin
*clec-45*	F07C4.2	90.52	C-type LECtin
*clec-78*	NM_068053	58.48	C-type LECtin
*clec-174*	Y46C8AL.2	36.26	C-type LECtin
*clec-46*	F07C4.9	22.51	C-type LECtin
*clec-196*	F26D10.12	21.08	C-type LECtin
**F-box protein-related**			
*fbxc-57*	C32B5.12	5.58	F-box C protein
*fbxb-37*	T16A1.8	32.25	F-box B protein
*fbxc-24*	R07C3.11	30.11	F-box C protein
*fbxb-70*	ZK909.5	26.53	F-box B protein
*fbxb-101*	Y63D3A.3	24.17	F-box B protein
*fbxb-53*	F36H5.5	20.38	F-box B protein
**NHR family-related**			
*nhr-172*	C54F6.9	26.72	Nuclear Hormone Receptor family
*nhr-127*	T13F3.3	21.42	Nuclear Hormone Receptor family
*nhr-221*	T24A6.8	15.07	Nuclear Hormone Receptor family
*nhr-25*	F11C1.6a.1	9.54	Nuclear Hormone Receptor family
*nhr-150*	C06B8.1	9.29	Nuclear Hormone Receptor family
*nhr-165*	C47F8.2	6.07	Nuclear Hormone Receptor family
*nhr-209*	R07B7.16	5.74	Nuclear Hormone Receptor family
**Hypothetical protein**			
*F48F5.3*	—	60648.71	hypothetical protein
*Y75B8A.7*	—	706.94	hypothetical protein
*Y41D4B.26*	—	366.72	hypothetical protein
*K08B4.2*	—	68.29	hypothetical protein
*C54C8.12*	—	53.99	hypothetical protein
*F47G4.6*	—	50.71	hypothetical protein
*F46B3.1*	—	42.03	hypothetical protein
*T04A6.2*	—	39.39	hypothetical protein
*F48F5.3*	—	60648.71	hypothetical protein

### Host response of *C. elegans* conditioned with JDFM216 involves NHR-25, NHR-127, and NHR-209 signaling pathways

**Figure 4:**
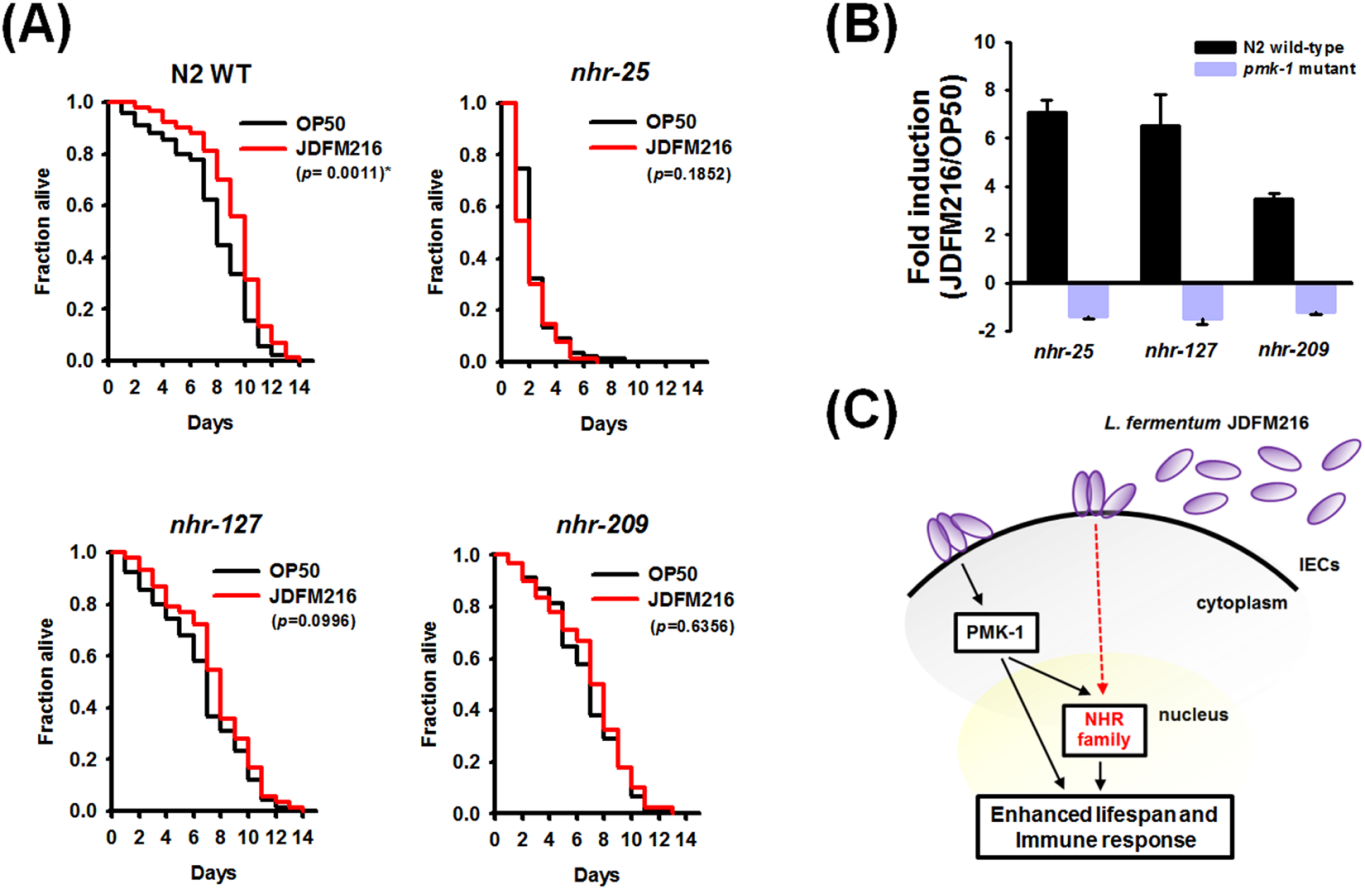
(**A**) NHR family is required for regulation of aging in *C. elegans* by *Lactobacillus fermentum* strain JDFM216. Lifespan assay of *C. elegans* strain N2 wild-type worms and loss-of function mutants including *nhr-25*, *nhr-127*, or *nhr-209* [Survival statics: wild-type N2 (p = 0.0011 worms conditioned with JDFM216 compared to OP50), *nhr-25* (p = 0.1852 worms conditioned with JDFM216 compared to OP50), *nhr-127* (p = 0.0996 worms conditioned with JDFM216 compared to OP50), and *nhr-209* (p = 0.6356 worms conditioned with JDFM216 compared to OP50)]. (**B**) qRT-PCR analysis evaluating the regulation of selected NHR genes associated with PMK-1 signaling. Transcript levels were measured in young adult N2 wild-type or *pmk-1* mutant worms conditioned with JDFM216 for 24 h. (**C**) Schematic mechanisms of enhanced lifespan and immune response of *L. fermentum* strain JDFM216 via NHR family and PMK-1 pathway.

To further investigate whether the NHR family contributes to nematode host defense-mediated JDFM216, we performed life span assays with loss-of-function mutants or N2 wild-type worms to confirm that the NHR genes are directly involved in the host defense of *C. elegans* preconditioned with JDFM216. As shown in Fig. 4A, compared with wild-type N2 fed with OP50, those fed with JDFM216 exhibited a significantly extended life span (p = 0.0011). Conversely, JDFM216 had no effect on life span of worms with deleted *nhr-25*, *nhr-127*, or *nhr-209* genes (p = 0.1852 for *nhr-25*, p = 0.0996 for *nhr-127*, and p = 0.6356 for *nhr-209*) (Fig. 4A). In particular, worms lacking *nhr-25* were more susceptible to aging than other mutants or N2 wild-type, even though they were preconditioned with JDFM216. NHR-25 is a known member of the widely conserved FTZ-F1 family of nuclear receptors, which are transcribed during embryonic and larval development^29^. The absence of *nhr-25*, but not of *nhr-127* and *nhr-209*, in the host *C. elegans* altered its longevity. Taken together, these results indicated that specific stimulation of NHR family genes, including *nhr-25*, *nhr-127*, and *nhr-209*, were associated with nematode responses to aging and immunity after conditioning with JDFM216. Lastly, to elucidate the new JDFM216-stimulated pathways involving PMK-1 signaling and NHR family interactions, we investigated the regulation of selected NHR family genes, including *nhr-25*, *nhr-127*, and *nhr-209*, in N2 wild-type or *pmk-1* mutants after conditioning with JDFM216. As expected, qRT-PCR results showed that transcriptional levels of *nhr-25*, *nhr-127*, or *nhr-209* were significantly affected by the exposure of JDFM216 in N2 wild-type (consistent with DNA microarray analysis results), whereas these phenotypes were abolished by the loss-of-function *pmk-1* (Fig. 4B). Therefore, we suggested that the enhanced longevity and immunity of *C. elegans* by JDFM216 are associated with the regulation of the NHR family as well as the PMK-1 signaling pathway (Fig. 4C).

## Discussion

Probiotics exert their benefits through immunomodulation, release of metabolites, or attachment to host cells^30^. Among the most widely used probiotics are lactic acid bacteria, particularly *Lactobacillus* spp., which are commonly found in humans as commensal microorganisms, making them favorable candidates for probiotic research^31^. In particular, it has recently been reported that *L. fermentum* benefits body composition and metabolism in humans and mice^32,33^, acts as a therapeutic agent against pathogenic strains^34^, and is used to treat gastrointestinal and respiratory illnesses^35^. In addition, *L. fermentum* strain LP3 and LP4 fulfill sufficient criteria to be used a starter culture probiotic in the production of cheese and other dairy products^36^. *C. elegans* is a useful *in vivo* host model to research probiotic bacteria–host interactions^37^. Aging is a complex process driven by diverse signaling pathways, and a few anti-aging pathways have been established. When modeling the effects of probiotics using *C. elegans*, it is important to distinguish between the effects of nutrition on life span as well as life span extension caused by the induction of host responses. We have previously reported that *Bacillus licheniformis* isolated from traditional Korean foods is a probiotic strain that is generally recognized as a safe strain that enhances the life span of *C. elegans* via host serotonin signaling^38^. Consistent with our findings, studies on *C. elegans* have shown a beneficial effect in the life span of nematodes grown on some lactobacilli strains^2^.

Several immune response signaling pathways have been identified in *C. elegans*^8^. Among them, PMK-1 signaling is a key feature of the *C. elegans* innate immunity. In this study, we also indicated that the PMK-1 pathway is involved in the probiotic effects of JDFM216 (Fig. 3). Our group has previously indicated that feeding *C. elegans* with *L. acidophilus* strain NCFM has a protective effect against the gram-positive pathogens *Enterococcus faecalis* and *S. aureus* via the PMK-1 pathway^17^. However, there are several differences in the mechanism of probiotic activity. Compared with JDFM216, *L. acidophilus* NCFM did not colonize on the *C. elegans* intestine and their probiotic impact might have been caused by the production of MAMPs or damage-associated molecular patterns and not by intact cells. In addition, JDFM216 enhanced the susceptibility of *C. elegans* to infection by Gram-positive and Gram-negative pathogens (Fig. 1B). However, NCFM and JDFM216 strains share common features with PMK-1 signaling. Therefore, we proposed that PMK-1 signaling is stimulated by multiple probiotic factors, including bacteria or host-derived molecules or intact cells.

A recent whole transcriptome analysis of microarray data has revealed differential gene expression during infection and colonization of *C. elegans* by a range of microbes as well as its growth^39^. Transcriptional regulation provides a major mechanism of metabolic network control, and nutrient-induced metabolic gene expression changes have been observed in organisms from bacteria to humans. Importantly, NHR is a transcription factor that directly binds to DNA and regulates the expression of specific genes involved in development, homeostasis, and metabolism^6^. Some metabolites interact directly with NHR to modulate their function, such as vitamin A that activates the retinoic acid receptor, vitamin D that activates the vitamin D receptor, and free fatty acids and eicosanoids that bind to peroxisome proliferator-activated receptor alpha. It is established that the NHR family is associated with longevity in humans and animals as well as *C. elegans*. However, few studies have attempted to comprehensively identify endogenous NHR ligands, and their specific roles or target genes in mechanisms underlying human longevity remain largely unclear. The mammalian NHR has demonstrated that even small changes in ligand structures can strongly affect gene transcription^40^. Interestingly, metabolic diseases in old age depend on NHR function, and the life span of *C. elegans* is influenced by DAF-12, an NHR^41^. Liu *et al*.^42^ have reported that DAF-12 negatively regulates the defense against pathogens via the downstream *let-7* family of microRNAs, which directly target SKN-1, a gene downstream of PMK-1. Similarly, our quantitative real time-polymerase chain reaction and assays on aging of mutant worms demonstrated that NHR family genes, including *nhr-25*, *nhr-127*, or *nhr-209*, are important partners that crosstalk with the PMK-1 pathway and may modify various behaviors, such as aging and immunity of *C. elegans*.

In conclusion, our results demonstrated that JDFM216 has beneficial effects on longevity and immunity of *C. elegans* and that this strain gets highly adhered to the worm’s intestine. We found that JDFM216 significantly stimulates *C. elegans* longevity and host defenses through NHR family-related transcriptions. Moreover, this interpretation is strengthened by the finding that this regulation is associated with an enhanced PMK-1 signaling. Taken together, our findings suggested that a potential probiotic isolate, JDFM216, can be used in health functional foods and therapeutic dietary supplements especially for metabolic disease and aging. Furthermore, studies are ongoing to develop a more sophisticated understanding of host aging and health via JDFM216 in mouse models. In addition, it has been established that extension of lifespan is associated with differential effects on morbidity onset, thus affecting healthspan in different ways^43^; hence, next studies are needed to evaluate the functionality of JDFM216 on the healthspan. These insights can lead to probiotic nutritional intervention to improve health and aging by target specific transcriptions.

## Methods

### Bacterial strains and culture conditions

JDFM216 used in this study was isolated from the feces of a Korean infant and was selected by screening of potential probiotic bacteria using *in vitro* assays. This strain was cultured in De Man, Rogosa, and Sharpe (MRS) broth (Difco, Sparks, MD) at 37 °C for 24 h. *Staphylococcus aureus* RN6390 was used as the pathogenic bacteria and grown at 37 °C in tryptic soy broth medium (BD Biosciences, Sparks, MD, USA). Another food-borne pathogen, *Escherichia coli* EDL933, was cultured at 37 °C in Luria–Bertani (LB) broth (BD Biosciences, Sparks, MD, USA). As standard feed for nematodes, *E. coli* strain OP50 (referred to as OP50) was grown in LB broth at 37 °C for 24 h with shaking (225 rpm)^17^. For long-term storage, cultures were maintained at −80 °C with 15% glycerol as a cryoprotectant. All strains were subcultured twice prior to experimental analysis.

To prepare live bacterial lawns for *C. elegans* feeding, bacteria were collected by centrifugation at 13,000 rpm for 1 min, washed twice with sterile M9 buffer, and centrifuged at 13,000 rpm for 1 min to remove the supernatant. The bacteria were adjusted to a final concentration of 2.5 mg (wet weight) per μL in M9 buffer and were subsequently used as a concentrated bacteria supply. The suspended cells were plated on nematode growth medium (NGM) plates and dried.

### *C. elegans* culture conditions

*C. elegans* N2 Bristol wild-type and mutant strains, including CF512 *fer-15(b26)II;fem-1(hc17)IV* (*fer-15;fem-1* worms), MH1955 *nhr-25(ku217)X*, VC1283 *nhr-127(gk572)V*, VC2567 *nhr-209(gk1135)V*, and KU25 *pmk-1(km25)IV* were obtained from the Caenorhabditis Genetics Center (St. Paul, MN, USA)^15,44^. For killing assay experiments, the *fer-15;ferm-1* mutants were used because they are unable to produce progeny at 25 °C without alteration in the *C. elegans* phenotype^17^. Worms were routinely maintained on NGM agar using standard techniques^45^ and were seeded with OP50, an internationally established feed. Eggs were extracted in sodium hypochlorite–sodium hydroxide solution from egg-bearing worms and then transferred onto NGM plates seeded with OP50. Synchronized L1 worms were grown at a restrictive temperature (25 °C) to obtain sterile L4/young adult worms.

### *C. elegans* life span and killing assays

To evaluate whether the candidate probiotics had an effect on the host life span, nematode life spans were determined using established methods with modifications^17^. Briefly, 100-μL aliquots of concentrated bacteria were plated on 35-mm-diameter NGM agar plates, and L4/young adult worms of N2 wild-type or mutant nematodes were individually transferred with a platinum wire onto OP50 or JDFM216 plates and were maintained at 25 °C. For each life span assay, 90 worms per bacterial species were assayed in three plates (30 worms per plate). The plates were incubated at 25 °C, and the numbers of live worms were measured daily for viability using an Olympus SZ40 dissection microscope. Nematodes were transferred to fresh plates daily during the progeny production period and, thereafter, were transferred every 3 days. A worm was determined to be alive or dead by gently touching with a platinum wire pick.

The *C. elegans* killing assay was performed as previously described^17^. L4/young adult worms were placed on conditioning plates with JDFM216 or OP50 at 25 °C until they were used for experiments on days 1, 3, 6, or 9 of adulthood. Subsequently, pathogen lawns for survival assays were prepared by inoculating modified NGM with an overnight bacterial culture. Nematodes preconditioned with JDFM216 or OP50 were transferred onto *S. aureus* RN6390 or *E. coli* EDL933 pathogen plates and incubated at 25 °C, followed by transferring live worms onto fresh bacterial lawns and examining at 24-h intervals until all worms died.

### Bacterial attachment assay using plate counting and transmission electron microscopy (TEM)

The number of bacteria in the nematode intestine was measured according to an established method^3^ with some modifications. After exposing worms for 24 h to JDFM216 lawns prepared on NGM agar plates, 10 worms were randomly picked, washed twice in M9 buffer, and transferred onto a brain–heart infusion agar plate containing kanamycin (100 μg/mL) and streptomycin (100 μg/mL). The worms were washed with 5-μL drops of gentamicin solution (25 μg/mL) for 5 min to remove surface-attached bacteria. Next, worms were washed five times with M9 buffer, lysed in M9 buffer with 1% Triton X-100, and mechanically disrupted using a mortar and pestle (Kontes, Vineland, NJ, USA). The worm lysates were serially diluted in M9 buffer and incubated overnight at 37 °C on modified MRS (pH 5.0) plates. Colonies were quantified and used to calculate the number of bacteria per nematode.

Simultaneously, bacterial attachment was visualized by TEM (HITACHI H-7650, Tokyo, Japan), as previously described^8^, to determine their persistence on the *C. elegans* intestinal epithelium. Worms were plated on 60-mm NGM plates seeded with JDFM216 or control plates seeded with OP50. For each observation, at least 10 cross-sections were evaluated, and representative images were chosen.

### RNA isolation and transcriptome analysis

Synchronized populations of *fer-15;fem-1* mutants were harvested to examine gene expression in the host in response to preconditioning with JDFM216 or OP50 for 24 h. Total RNA from worms was immediately isolated using TRIZOL reagent (Invitrogen), according to the manufacturer’s instructions, and purified using an RNeasy Mini Kit (Qiagen) including an on-column DNase digestion with RNase-free DNase (Qiagen) coupled with a mini bead beater (Biospec, Bartlesville, OK). The synthesis and fragmentation of target cRNA probes and hybridization were performed using Agilent’s Low RNA Input Linear Amplification kit (Agilent Technology, Santa Clara, CA), according to the manufacturer’s instructions. The fragmented cRNA was resuspended in 2×hybridization buffer and then applied with a pipette directly onto *C. elegans* 4 × 44 K microarray chip (Agilent Technology). The arrays were hybridized at 65 °C for 17 h using Agilent’s Hybridization Oven. The hybridized microarrays were washed according to the manufacturer’s protocol (Agilent Technology) and were analyzed using GenePix Pro 6.0 (Axon Instruments, Foster City, CA). The average fluorescence intensity for each spot was calculated, and local background was subtracted. All data normalization and selection of fold-changes were performed using GeneSpring 7.3.1 (Agilent Technology). Intensity-dependent normalization [locally weighted regression scatter plot smoothing (LOWESS)] was performed, wherein the ratio was reduced to the residual of the LOWESS fit of the intensity versus ratio curve. Averages of the normalized ratios were calculated by dividing the average normalized signal channel intensity by the average normalized control channel intensity. A gene was considered differentially expressed when the p-value for comparing two chips was <0.05 (to ensure that the change in gene expression was statistically significant and that false positives were <5%). Functional annotations of genes from the Gene Ontology Consortium were retrieved with GeneSpring GX 7.3. Genes were based on searches of DAVID (http://david.abcc.ncifcrf.gov/) and Medline (http://www.ncbi.nlm.nih.gov/) databases.

### Quantitative real-time PCR (qRT-PCR)

qRT-PCR was performed using the StepOne™ Real-Time PCR System (Applied Biosystems, Foster City, CA). Fifty microgram of total RNA were used for the qRT-PCR reaction using the SuperScript^TM^ III Platinum^®^ SYBR^®^ Green One-Step qRT-PCR Kit (Invitrogen). Primers were designed using Primer3Input Software (v0.4.0) and are listed as follows: *nhr-25* (5′-TCTTGGCGTGAAGTGAGATG-3′ and 5′-AGAATTGCTCCCAGTTCGTG-3′), *nhr-127* (5′-GGAAGCCGACTGAAATTGAA-3′ and 5′-CCGAAAAGCGTAATTTTCCA-3′), *nhr-209* (5′-ATAGGTGGATGCTTGGCTTC-3′ and 5′-TTGCATATTGGTTCCGTTCA-3′), and *snb-1* (5′- CCGGATAAGACCATCTTGACG-3′ and 5′-GACGACTTCATCAACCTGAGC-3′). Relative expression levels were calculated using the 2^−ΔΔCt^ method. The control gene *snb-1* was used to normalize the genes expression data.

### Immunoblot analysis

*C. elegans* were preconditioned with JDFM216 or OP50 for 24 h. After treatment, the worms were washed with M9 buffer and homogenized in lysis buffer, RIPA buffer (25 mM Tris pH 7.6, 150 mM NaCl, 1% NP- 40, 1% sodium deoxycholate, and 0.1% sodium dodecyl sulfate [SDS]) supplemented with cOmplete^TM^ Mini protease inhibitor cocktail (Roche) and phosphatase inhibitor cocktail (Sigma-Aldrich). The residues in the lysates were removed by centrifugation at 13,000 × g for 10 min at 4 °C, and protein content was measured. Proteins were loaded on to 12% reducing SDS-acrylamide gels and electrophoresis, and transferred to polyvinylidenefluoride (PVDF) membrane of 0.2 μm pore size. After transfer, the membranes were blocked with 1% bovine serum albumin and 5% skimmed milk in Tris-buffered saline containing 0.1% Tween 20 (TBS-T) for 2 h and incubated 1 h at room temperature with primary antibody as follows: Phospho-PMK-1 (Cell Signaling) or β-actin (Santa cruz). Membranes were washed with TBS-T and incubated for 1 h at room temperature with horseradish peroxidase (HRP)-conjugated secondary antibody as follows: anti-Rabbit IgG (Thermo Fisher) or anti-mouse IgG (Santa cruz). HRP signals were visualized using the chemiluminescence (ECL kit, Amersham) and an image analyzer (C-DiGit® Blot Scanner, Li-COR).

### Statistical analysis

*C. elegans* survival analysis was performed using the Kaplan–Meier method, and the significance of differences between survival curves was determined using log-rank test (STATA6; STATA, College Station, TX, USA). Student’s *t* test was performed to determine statistical differences for CFU counting for determining bacterial abundance. All data represent the results of three independent replicates. A p-value of 0.05 relative to control in all replicate experiments was considered to reflect a significant difference from the control.

## Electronic supplementary material

Supplementary information
