# Supplementary material for: Probiotic Lactobacillus fermentum strain JDFM216 stimulates the longevity and immune response of Caenorhabditis elegans through a nuclear hormone receptor
Source: Sci Rep. 2018 May 10;8:7441. doi: 10.1038/s41598-018-25333-8 (PMC5945636; doi:10.1038/s41598-018-25333-8)
Supplement: Supplementary file 1 — Supplementary information [file 41598_2018_25333_MOESM1_ESM.docx]

Supporting Information

Probiotic *Lactobacillus fermentum* strain JDFM216 stimulates the longevity and immune response of *Caenorhabditis elegans* through a nuclear hormone receptor

Mi Ri Park^1^, Sangdon Ryu^1^, Brighton E. Maburutse^1^, Nam Su Oh^2^, Sae Hun Kim^3^, Sejong Oh^4^, Seong-Yeop Jeong^5^, Do-Youn Jeong^5^, Sangnam Oh^6†^, and Younghoon Kim^1†^

^1^Department of Animal Science and Institute of Milk Genomics, Chonbuk National University, Jeonju, 54896, Korea

^2^R&D Center, Seoul Dairy Cooperative, Ansan, Gyeonggi 15407, South Korea

^3^Department of Biotechnology, College of Life Sciences and Biotechnology, Korea University, Seoul 02841, South Korea

^4^Department of Animal Science, Chonnam National University, Gwangju 61186, Korea

^5^Microbial Institute for Fermentation Industry, Sunchang, Jeonbuk 56048, Republic of Korea

^6^Department of Functional Food and Biotechnology, Jeonju University, Jeonju 55069, Republic of Korea

**Supplementary Figures.**

Supplementary Figure S1.

Supplementary Figure S2.

Supplementary Figure S3.

**Supplementary Table**

Supplementary Table S1.


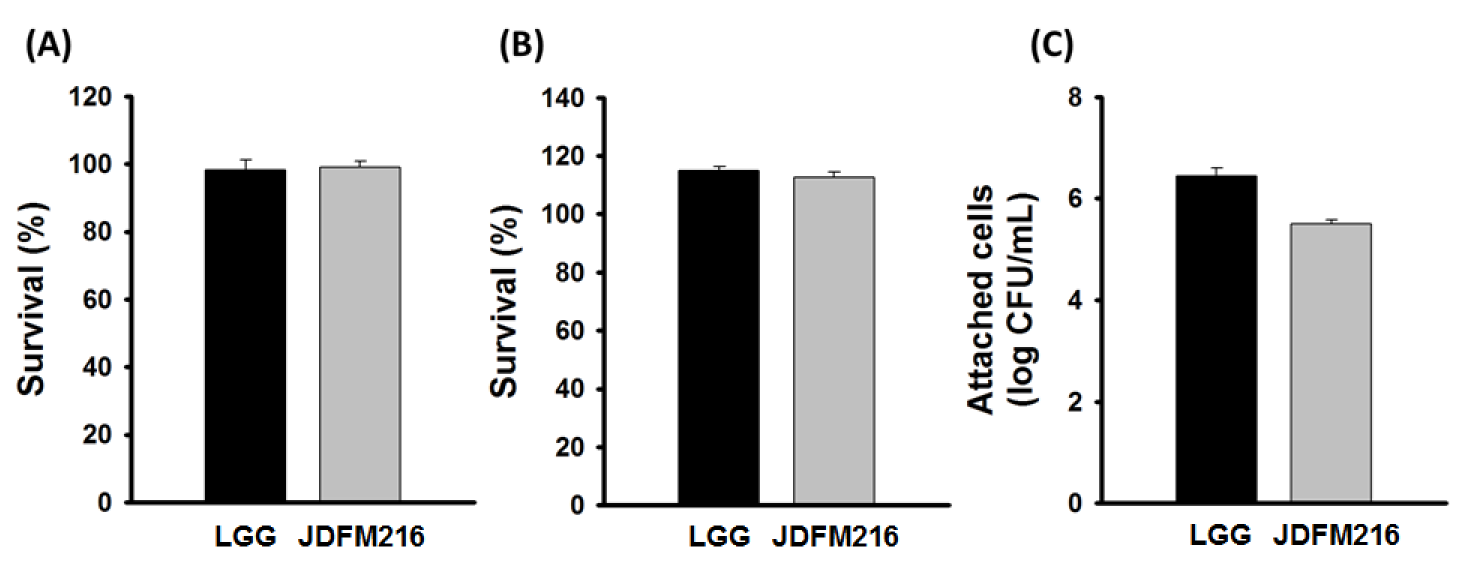


**Figure S1.** Conventional in vitro assay results for *Lactobacillus fermentum* strain JDFM216. Susceptibility of JDFM216 to (A) synthetic gastric juice (pH 2.5) and (B) bile conditions with 0.5% oxgall as well as their ability for (C) attachment on a mucus layer *in vitro*. Values represent the mean of three independent experiments with standard error of the mean (SEM). LGG = *Lactobacillus rhamnosus* strain GG.

**
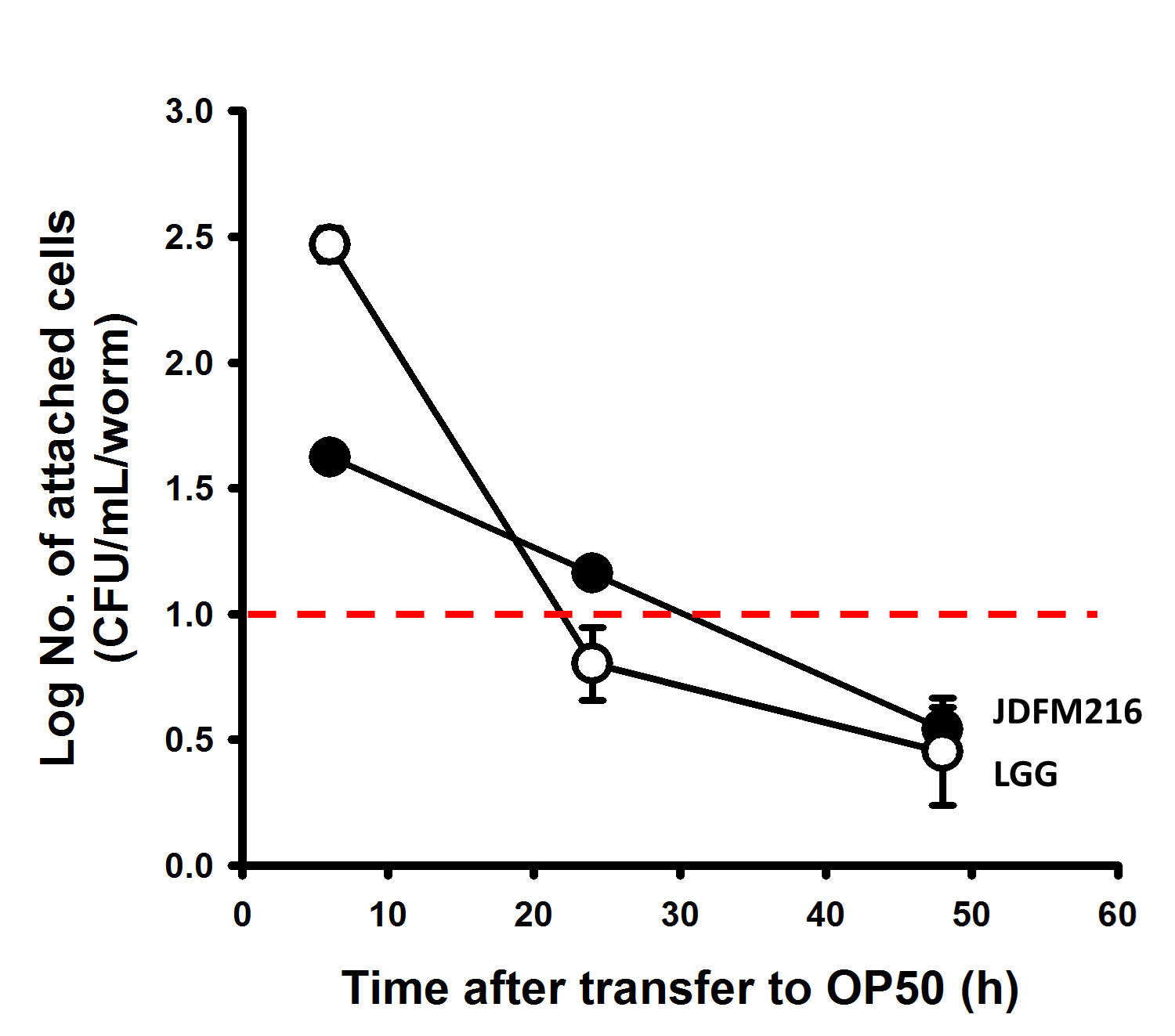
**

**Figure S2.** Quantification of intestinal *Lactobacillus fermentum* strain JDFM216 obtained by disruption of worms feeding on OP50 at 6, 24, and 48 h after 24 h of feeding on JDFM216. Values represent the mean of three independent experiments with standard error of the mean (SEM). LGG = *Lactobacillus rhamnosus* strain GG.

**
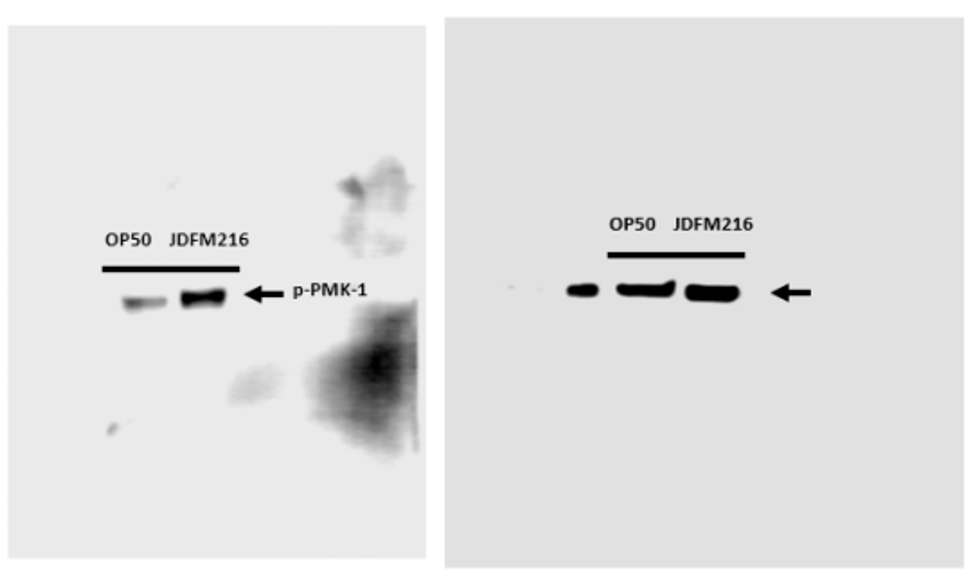
**

**Fig. S3.** Uncropped images of western blot for all panels shown in Fig. 3D.

**Table S1.** Antimicrobial activities of *Lactobacillus fermentum* strain JDFM216 on four foodborne pathogens.

| Pathogenic strains | Diameter of inhibition zone (mm) |
| --- | --- |
| *E. coli* O157: H7 EDL 933 | 26.71±1.14 (++) |
| *Salmonella* Typhimurium SL1344 | 28.05±1.57 (++) |
| *Listeria monocytogens* EGD-e | 23.52±3.31 (+) |
| *Listeria monocytogens* Scott A | 24.71±1.76 (+) |

Inhibition zone: 0 mm no activity, <5 mm: 5-20 mm: Weak (±), 20-25 mm: Moderate (+), ≥25 mm: Strong (++), Mean values with standard deviation (n = 3).
